# Unleashing the Power of IL-17: A Promising Frontier in Chronic Obstructive Pulmonary Disease (COPD) Treatment

**DOI:** 10.7759/cureus.41977

**Published:** 2023-07-16

**Authors:** Christine Henen, Elise A Johnson, Shimshon Wiesel

**Affiliations:** 1 Internal Medicine, St. John's Episcopal Hospital, New York, USA; 2 Medical School, Ross University School of Medicine, Miramar, USA; 3 Pulmonary/Critical Care/Sleep Medicine, St. John's Episcopal Hospital, New York, USA

**Keywords:** – pulmonary hypertension, β-catenin pathway, biologic agents, copd (chronic obstructive pulmonary disease), il17

## Abstract

Chronic obstructive pulmonary disease (COPD) is a pulmonary ailment that is both degenerative and incapacitating, with a global prevalence affecting millions. Despite notable progress in treatment methodologies, there is still a critical requirement for innovative therapeutic interventions. The pathogenesis of COPD has recently seen a significant focus on the role of interleukin 17 (IL-17), a pro-inflammatory cytokine. This review investigates the potential of IL-17 targeting as a viable therapeutic approach for treating COPD. The literature indicates a complex correlation between IL-17 and COPD. Research has indicated that IL-17 plays a role in the manifestation of airway inflammation, remodeling, and mucus hypersecretion, considered characteristic attributes of COPD. Elevated levels of IL-17 have been observed in the lungs of individuals with COPD, indicating its potential as a therapeutic target for intervention. Furthermore, preclinical studies utilizing animal models of COPD have demonstrated the efficacy of anti-IL-17 antibodies in reducing airway inflammation and remodeling. Comprehending the mechanical principles that underlie IL-17 signaling in COPD is imperative for advancing focused, therapeutic interventions. Activating diverse signaling pathways, such as the β-catenin and Act 1 adaptor protein (ACT 1) mediated pathways, is a crucial aspect of COPD pathogenesis triggered by IL-17. As a result, the suppression of IL-17 signaling has exhibited encouraging outcomes in mitigating pulmonary hypertension induced by hypoxia and interrupting the signaling mediated by ACT 1. Notwithstanding these promising discoveries, additional investigation is required to comprehensively explain the function of IL-17 in COPD and its viability as a target for therapy. The efficacy and safety of biological treatments that target IL-17 in COPD patients necessitate thorough investigation despite their initial positive outcomes. Furthermore, identifying appropriate patient subpopulations that would benefit most from IL-17-targeted therapies and optimizing treatment protocols are binding domains for future investigation. The current review presents a persuasive case for the imperative requirement of an additional investigation into the targeting of IL-17 for COPD management. Through a comprehensive analysis of the complex relationship between IL-17 and COPD pathogenesis, novel therapeutic avenues can be explored, potentially transforming the approach to managing this incapacitating condition. As we explore this novel domain, the possibility of pioneering therapies aimed at IL-17 presents a ray of optimism for the multitudes of individuals afflicted with the onerous consequences of COPD.

## Introduction and background

Chronic obstructive pulmonary disease (COPD) is a significant health challenge, affecting many  individuals globally and substantially burdening their well-being. With the increasing prevalence of COPD, it is imperative to investigate novel therapeutic strategies to address this persistent ailment. In contemporary times, there has been a shift in emphasis toward interleukin 17 (IL-17),  a pro-inflammatory cytokine, as a promising candidate for transforming the treatment of COPD [1]. This review aims to elucidate the pivotal function of biological interventions that target IL-17 and underscore the necessity for additional investigation in this promising domain. COPD's pathogenesis is a multifactorial and intricate process encompassing  chronic inflammation, progressive tissue destruction, and airflow limitation. The role of IL-17 in the inflammatory pathway linked to COPD has been identified as substantial. Multiple research investigations have demonstrated elevated concentrations of IL-17 within the respiratory system of COPD patients, which is linked to the gravity and progression of the ailment. Furthermore, preclinical models have provided compelling evidence regarding the harmful effects of IL-17, including airway inflammation, remodeling, and excessive mucus production [2].

The strategic focus on IL-17 presents a significant opportunity for revolutionizing the COPD therapeutic landscape. Preclinical studies have demonstrated promising outcomes in reducing airway inflammation and attenuating disease-related symptoms by inhibiting IL-17 signaling pathways [3]. The employment of Anti-IL-17 antibodies has emerged as a promising therapeutic strategy, affording the possibility to directly counteract the effects of IL-17 and mitigate its harmful outcomes [3]. Even with  the encouraging results, additional investigation is imperative to elucidate the complex mechanisms that underlie the role of IL-17 in the pathogenesis of COPD. Comprehending the exact mechanisms of signal transduction and subsequent outcomes of IL-17 is imperative in advancing  efficacious and securely directed treatments [4]. Identifying  appropriate patient subgroups most likely to benefit from IL-17-targeted therapies is paramount.

The present analysis examines the current corpus of scholarly works, exploring the potential therapeutic prospects of targeting IL-17 in COPD [5]. Through  synthesizing evidence from multiple studies, our objective is to elucidate the potential of biological treatments and catalyze additional research in this promising domain. By thoroughly examining  the existing literature, we aim  to identify areas of insufficient understanding, suggest potential directions for further investigation, and inspire scholarly interest in advancing the frontiers of COPD therapy.

## Review

Methodology

A comprehensive literature search was performed to investigate the potential of IL-17 as a therapeutic target. The selection of pertinent articles was based on their emphasis on IL-17 and its correlation with the pathogenesis and treatment of COPD. The investigation involved the exploration of various databases, including PubMed, through the utilization of specific keywords such as "IL-17," "biologic treatments," "COPD," and other related terminologies. The chosen articles underwent a rigorous critical evaluation process to extract significant findings and insights about potential therapies targeting IL-17 for treating COPD. The included studies discussed diverse methodologies, encompassing in vitro experiments, animal models, clinical trials, and reviews.

Review

The study by Wang et al. examined the effects of IL-17 on pulmonary hypertension induced by hypoxia. The findings of their investigation suggest that the amelioration of pulmonary hypertension can be accomplished by targeting IL-17, resulting in the downregulation of β-catenin. Their study indicates that inhibiting IL-17 could be viable for managing vascular complications commonly linked with COPD [6]. Roos and Stampfli investigated the mechanical concepts and therapeutic opportunities associated with IL-17 signaling in lung disease induced by cigarette smoke. They emphasized the importance of IL-17 in neutrophilic inflammation and airway remodeling, proposing it as a potential target for therapeutic intervention in treating COPD [2]. Kramer and Gaffen examined the function of IL-17 in the context of inflammation, autoimmunity, and its possible therapeutic applications. This groundbreaking study laid the groundwork for understanding the role of IL-17 in various pathological states, including COPD [1]. In a study conducted by Shen et al., it was observed that anti-IL-17 antibodies were efficacious in mitigating airway inflammation in mice exposed to tobacco smoke. The findings of their investigation suggest that targeting IL-17 through interventions could serve as a viable approach to mitigate the negative ramifications of smoking in COPD patients [7]. In general, while the present study on biological interventions targeting IL-17 in COPD displays potential, it is crucial to conduct further inquiry to address the current gaps in knowledge and enhance the therapeutic effectiveness. Subsequent research endeavors ought to clarify the exact mechanisms underlying the role of IL-17 in the pathogenesis of COPD. 

Additionally, it is imperative to identify particular patient subgroups that would derive the greatest benefit from IL-17-targeted therapies. In light of this, it is crucial to devise novel strategies to surmount potential obstacles and adverse effects [8]. Perhaps, it is imperative to subject IL-17-targeted biologic treatments to rigorous evaluation through well-designed clinical trials to assess their clinical applicability and long-term safety. Moreover, an all-encompassing comprehension of the interactions between IL-17 and other molecular pathways and its function in various stages and phenotypes of COPD would establish a firm basis for customized therapeutic interventions. 

Discussion 

The investigation of IL-17's involvement in the pathogenesis of COPD is a topic of academic significance. IL-17 has been identified as a significant factor in the pathogenesis of COPD [1]. Roos and Stampfli conducted a study that indicates a correlation between exposure to cigarette smoke and increased levels of IL-17 in the pulmonary system of individuals with COPD [2]. The upregulation of IL-17 expression has been associated with increased airway inflammation and tissue damage, thereby contributing to the pathogenesis of COPD. Emerging evidence indicates that IL-17 and interleukin-22 (IL-22), which play a crucial role in regulating pulmonary inflammation and infection, are implicated in COPD pathophysiology [2]. Shen et al. posited that the progression and exacerbation of the disease could be ascribed to an increase in the secretion of IL-17, which leads to the recruitment of neutrophils, ultimately resulting in persistent inflammation, airway obstruction , and emphysema [7]. During the established phase of COPD, a compromised IL-22 response contributes to infections associated with pathogens and exacerbations of the disease. The dysregulation of cytokine production is rooted in the complex interplay between immune cells and antigen-presenting cells that are not functioning properly. Shen et al. have reported that the upregulation of IL-17 expression leads to the release of supplementary inflammatory mediators such as interleukin-8 (IL-8) and tumor necrosis factor-alpha (TNF-α), thereby augmenting the inflammatory response [7]. Hence, the precise and deliberate focus on IL-17 signaling can potentially be a highly effective therapeutic approach in reducing the chronic inflammation that characterizes COPD. 

Mechanistic Insights Regarding IL-17 Signaling

Comprehending the mechanisms that underlie the signaling of IL-17 is paramount in developing efficacious biologic treatments. Roos and Stampfli offered mechanistic elucidation about the signaling of IL-17 in lung disease induced by cigarette smoke [2]. The authors underscored the significance of IL-17 in the context of neutrophilic inflammation and airway remodeling, highlighting its potential as a promising therapeutic target. Additionally, research has indicated that IL-17 operates via the ACT1-mediated signaling pathway [4]. Using inhibitors such as pevonedistat (MLN4924) can protect against pulmonary inflammation induced by IL-17A by disrupting the relevant pathway. IL-17A, a type of cytokine, has been suggested to be a strong supporter in protecting against invading pathogens that target exposed epithelial surfaces. The function of this tissue is to provide immune surveillance against microbial agents, thereby protecting the integrity of the barrier. According to Mammen et al., IL-17A is critical in safeguarding tissues against potential microbial threats and restoring epithelial homeostasis. Recruitment of effector cells to the site of inflammation is one of the mechanisms by which it achieves its function. Furthermore, IL-17A promotes the host's immune response by inducing the formation of abnormal lymphoid structures [8]. The existing literature emphasizes the noteworthy contribution of IL-17A in the pathogenesis and clinical manifestations of three autoimmune disorders, namely systemic lupus erythematosus, Sjögren's syndrome, and systemic sclerosis, which have the potential to cause mortality [1]. The aforementioned mechanical concepts serve as a fundamental basis for investigating the therapeutic capacity of IL-17 inhibition in the management of COPD.  

Vascular Complications in COPD

COPD has been observed to have an impact not only on the airways but also on the pulmonary vasculature. The study by Wang and colleagues aimed to examine the influence of IL-17 on pulmonary hypertension induced by hypoxia. The study's findings indicated that the mitigation of pulmonary hypertension was observed by targeting IL-17, which resulted in the downregulation of β-catenin [6]. The results of this study indicate that the inhibition of IL-17 may have wider implications beyond the inflammation of the airways, as it may also address the vascular complications linked to COPD.  

Engaging in a discourse regarding IL-17 as a pivotal modulator of inflammation is worthwhile. The cytokine IL-17 is known to have a pivotal function in coordinating the inflammatory reaction in COPD. The study conducted by Shen et al. provided evidence that the administration of anti-IL-17 antibodies was successful in reducing airway inflammation in mice exposed to tobacco smoke [7]. The discovery implies that biologics targeted towards IL-17 can mitigate inflammation and its adverse consequences in individuals with COPD. IL-17 can either facilitate innate immunity against pathogens or exacerbate the development of inflammatory disorders, such as rheumatoid arthritis and psoriasis. Additionally, the role of IL-17 in regulating the interactions between Mucin and Galectin-3 in asthma has been identified [8]. Given the observed overlap in their pathophysiology, the potential therapeutic benefits for asthma and COPD could be achieved by targeting IL-17. Clinical trials have demonstrated promising outcomes for psoriasis and rheumatoid arthritis through systemic treatments with anti-IL-17 biologics. Nevertheless, the impact of these therapies on the commonly occurring periodontal disease remains unexplored and undocumented. Further clinical trials are necessary to establish the involvement of IL-17 in periodontitis, with a preference for using locally administered IL-17 blockers [8]. Additionally, these trials are crucial in determining effective adjunctive treatment for this inflammatory disease of the oral cavity. The significance of IL-17 in regulating inflammation underscores its potential as a therapeutic target in COPD. 

Potential Clinical Implications and Biological Therapies Associated with IL-17 in COPD

Although preclinical studies offer encouraging findings, conducting clinical trials to investigate the effectiveness and safety of biological treatments that target IL-17 in individuals with COPD is imperative. Yousuf et al. examined the potential therapeutic applications of T2 biologics, specifically IL-17 inhibitors, for treating COPD. The authors emphasized the necessity for additional research to establish the most effective patient selection criteria and dosing regimens for therapies targeting IL-17. In broad terms, advancing biological interventions targeting IL-17 presents novel prospects for managing COPD. The potential of T2 biologics as a viable option for treating COPD was discussed with particular emphasis on therapies targeting IL-17 [3]. T2 biologics exhibit selective modulation of particular immune pathways, such as IL-17 signaling, to mitigate the persistent inflammation commonly observed in COPD [3]. Biologic agents have demonstrated potential in mitigating exacerbations, ameliorating pulmonary function, and augmenting the quality of life among individuals afflicted with COPD. However, additional investigation is required to enhance their effectiveness, establish the optimal patient demographic, and assess their safety over an extended period. To facilitate the translation of preclinical research outcomes into clinical practice, it is imperative to conduct meticulously planned clinical trials that evaluate the effectiveness, safety, and enduring consequences of biological interventions that target IL-17. The notion of a universal fit is only applicable in some situations. Or does it have an impact? Topical glucocorticoids have been dermatologists' preferred treatment option for most inflammatory skin conditions for over 50 years. These agents have demonstrated remarkable anti-inflammatory effects, providing prompt relief from itch and rash and restoring inflamed skin to a state that is almost normal within a few days [5].

The heterogeneity of COPD poses a challenge in the implementation of IL-17-targeted therapies. COPD comprises diverse phenotypes, such as emphysema-dominant and bronchitis-dominant forms, characterized by unique underlying pathophysiological mechanisms [9]. Hence, it is imperative to identify particular subpopulations of patients that would derive maximum advantages from inhibiting IL-17, thereby facilitating personalized healthcare [4,10]. The implementation of genetic and biomarker profiling has the potential to facilitate patient stratification and forecast treatment response. This approach could enable the administration of targeted therapy utilizing IL-17 inhibitors in individuals most likely to derive benefits.

Safety Protocols and Parameters of the Clinical Trials

The accurate assessment of IL-17-targeted therapies' safety profile in COPD is imperative. As with any biological intervention, performing a thorough assessment of possible adverse events and enduring outcomes is crucial. Assessing the safety and efficacy of IL-17 inhibitors in COPD necessitates conducting clinical trials. Desai and colleagues (2022) have suggested that an inhibitor targeting Retinoic Acid Receptor-related Orphan Receptor-gamma (RORγ), a crucial regulator of IL-17 production, could serve as a promising therapeutic option for COPD. The present investigation illustrates the continuous endeavors to create innovative therapies that target IL-17 and highlights the significance of meticulous clinical trials in determining their role in managing COPD [5]. 

Potential Future Directions Needed to Address Knowledge Gaps in IL-17-Targeted Therapies for COPD

Additional investigation is required to address various gaps in knowledge about therapies targeting IL-17 in the context of COPD. Clarifying the specific mechanisms through which IL-17 contributes to the pathogenesis of COPD would yield significant insights for therapeutic interventions. Moreover, a comprehensive comprehension of the interplay between IL-17 and other molecular pathways implicated in COPD may reveal innovative therapeutic targets and collaborative treatment strategies [4]. Subsequent research endeavors should investigate combining therapies involving IL-17 inhibition and other targeted interventions. A significant obstacle in managing COPD is the presence of heterogeneity, whereby distinct subtypes of patients may exhibit diverse reactions to IL-17 inhibition. Desai and colleagues have proposed that a RORγ inhibitor could be a viable therapeutic intervention for managing COPD. This alternative methodology may present novel opportunities for the modulation of IL-17. Subsequent investigations ought to prioritize the identification of biomarkers or phenotypic traits that have the potential to forecast the response of patients to therapies targeting IL-17. The resolution of these obstacles and the investigation of alternative methodologies will significantly contribute to the advancement of individualized therapies based on IL-17 for COPD [5]. A comprehensive understanding of the role of IL-17 in COPD can be attained by amalgamating the outcomes of basic scientific research with clinical observations. The facilitation of collaborative research endeavors can aid in translating preclinical discoveries with potential into efficacious therapeutic interventions for individuals afflicted with COPD. 

Safety and Side Effects of Some Available Biologic Agents Targeting IL-17

There are multiple mechanisms by which biologic agents inhibit inflammatory signaling. Ixekizumab and secukinumab inhibit IL-17A, bimekizumab inhibits IL-17A and IL-17F, and brodalumab inhibits IL-17 RA and IL-17 RC. Due to the differences in the mechanism of IL-17 inhibition amongst these biologics, there may be variations in the adverse events and safety profiles of these individual agents [11]. Overall, they have shown favorable safety profiles in clinical trials with the most common adverse effects including hypersensitivity reactions, upper respiratory tract infection, *Candida* infections, pruritus, diarrhea, cough, arthralgias, headache, back pain, and cough. More serious adverse events that have been associated with IL-17 inhibitions with biologics are neutropenia, malignant or unspecified tumors, major adverse cardiovascular events (MACE), and inflammatory bowel disease [12,13]. The true adverse event profiles of these drugs may not be entirely described by the clinical data that is currently available. There is a need for vigorous pharmacovigilance efforts in validating the safety profile of the agent as well as documenting and studying any new adverse event signals that are not recognized yet. Because IL-17 plays an important role in driving innate inflammatory response and maintenance of the mucocutaneous barrier, IL-17 inhibitors may increase the risk of infection, dermatological disease, integrated stress response, and inflammatory bowel disease. These more common adverse events can typically be managed with antimicrobials without discontinuation of the drug or alternatively switching to a different anti-IL-17 biologic, non-IL-17 biologic, glucocorticoid, or nonbiologic immunosuppressive agent. However, evidence regarding other adverse safety signals is quite scarce and requires long-term studies to establish definitive causal relationships and subsequent management. Furthermore, additional studies on vulnerable populations are imperative and the effect of these biologics on comorbidities needs to be investigated further to understand the true safety profile and make informed clinical decisions for prescribing these agents [14]. 

## Conclusions

In a nutshell, the pursuit of IL-17 targeting exhibits a promising therapeutic strategy for managing COPD. The reviewed literature underscores the role of IL-17 in the pathogenesis of COPD, which encompasses multiple factors, including inflammation, airway remodeling, and vascular complications. Comprehending the mechanisms underlying IL-17 signaling and the consequent development of biological therapies that target IL-17 represents a robust basis for future investigation. It is imperative to conduct clinical trials to assess the effectiveness, safety, and enduring consequences of IL-17 inhibitors to ascertain their potential for managing COPD. The development of personalized IL-17-based treatments can be facilitated by addressing challenges such as patient heterogeneity and promoting collaborative efforts. In general, targeting IL-17 as a treatment for COPD exhibits significant potential in ameliorating persistent inflammation and enhancing patient outcomes. The true adverse event profiles of these drugs may not be entirely described by the clinical data that is currently available. There is a need for vigorous pharmacovigilance efforts in validating the safety profile of the agent as well as documenting and studying any new adverse event signals that are not recognized yet. The role of IL-17 in the development of COPD, the potential methods for suppressing IL-17, and the therapeutic possibilities offered by IL-17-targeted biologics provide a strong foundation for further research. Enhancing the effectiveness and safety of therapies targeting IL-17 in COPD requires the integration of personalized medicine, safety precautions, and meticulously planned clinical trials. Through the exploration of these facets and the progression of our comprehension of IL-17 biology, we have the potential to establish novel therapeutic approaches and revolutionize the realm of COPD management. 
